# Dry versus Wet Particle Assembly: Toward Solvent-Free Fabrication

**DOI:** 10.1021/acsami.5c05262

**Published:** 2025-06-05

**Authors:** Ignaas S. M. Jimidar, Kai Sotthewes

**Affiliations:** † Department of Chemical Engineering CHIS, Vrije Universiteit Brussel, Brussels 1050, Belgium; ‡ Mesoscale Chemical Systems, MESA+ Institute, University of Twente, P.O. Box 217, Enschede 7500AE, Netherlands; § Physics of Interfaces and Nanomaterials, MESA+ Institute, University of Twente, P.O. Box 217, Enschede 7500AE, Netherlands

**Keywords:** solvent-free colloidal assembly, dry particle assembly, dry powder rubbing, colloidal assembly, ordered particle mono- and multilayers

## Abstract

For over three decades, the continuous demand for miniaturized devices has prompted scientists to explore the fundamental principles of colloidal or particle (10 nm to 10 μm) assembly to construct large ordered structures. So far, most of the assembly has been performed in wet conditions, i.e., in a solution. Over the past decade, solvent-free (or dry) assembly methods have gained more attention, offering a more sustainable alternative. In this perspective, we highlight the promising aspects of the dry assembly method, which is not only easy to use, rapidly performed, and clean but also abandons the use of solvents while ensuring that the quality of the created particle assembly matches or exceeds that of wet particle assemblies. However, challenges remain for ordered multilayers, binary layers, and nonspherical particle assemblies due to strong surface forces and limited control. Learning from strategies used in wet assembly and gaining a deeper fundamental understanding of surface interactions at the micro- and nanoscale could help bridge these gaps. In this perspective, the challenges and opportunities of dry particle assembly are thoroughly explored, providing a roadmap for advancing the solvent-free assembly field and contributing to a more sustainable world.

## Graphical abstract

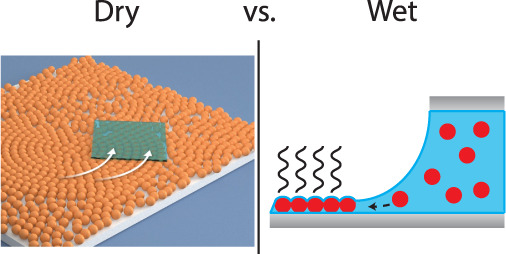

## 1 Introduction

Colloidal self-assembly involves the organization of nanometer- to micrometer-sized particles into secondary structures, where the collective properties are influenced not solely by the individual particle characteristics but also by the superstructure’s symmetry, orientation, phase, and dimensions. The fabrication of colloidal building blocks into assembled structures is integral to a wide range of applications, from studying fundamental phenomena such as phase transitions to practical uses in photonics, colloidal lithography, antibacterial surfaces, liquid chromatography, analytical spectroscopy devices, energy harvesting, sensors, cell culture substrates, and self-cleaning surfaces. Achieving well-ordered arrays relies heavily on manipulating or balancing surface interaction forces, and a complete comprehension of these surface interaction forces has been paramount in guiding scientists to develop a plethora of assembly methods over the past decades.

These surface interactions can be attractive or repulsive in nature, and their dominance is affected by wet and solvent-free environments. These surface interaction forces, including friction forces essential in dry assembly, have been extensively described in great detail in the literature^1–9^ and are, therefore, only broadly introduced here. These assembly forces include capillary, van der Waals, contact mechanics, electrostatics, hydrodynamics, and depletion interactions. The contact mechanics force is a mechanical force that emerges when two objects come into contact due to the deformation of the solids (depending on the Young’s modulus *Y*) involved. This force consists of compressive and adhesive components, which act perpendicular to the interface, along with frictional forces that influence movement in the tangential direction.^1,2^ The contact mechanics force has a dominating effect in dry assembly, whereas in a wet environment state, this force can be neglected. On the other hand, all the other interactions can be controlled during wet assembly by matching a solvent with a specific particle property. In addition, Brownian motion, sedimentation (influenced by gravity and buoyancy), ionic dissociation, and trapping particles at interfaces combined with lateral diffusion can all be exploited in wet assembly to attain ordered structures. Thus, scientists have a large “toolbox″ available to tune the interactions in wet environments, favoring wet colloidal assembly, i.e., particles suspended in a solution with techniques such as dip coating, solvent evaporation, Langmuir–Blodgett, drag coating, and drop casting.

A significant advantage of wet assembly techniques compared to dry methods in terms of surface interactions is the absence of capillary forces between the particles and the substrate. Capillary forces typically represent the strongest surface interactions^3,10^ hindering the controlled assembly under dry conditions. Additionally, the presence of the electrical double layer^11,12^ allows for easy tuning of surface forces, a feature not available in dry assembly methods. This control enables the formation of monolayers composed of single-type, binary, or even ternary particles in both two- and three-dimensional configurations.

The inevitable transition to a more sustainable industry in which solvent consumption is reduced and fabrication processes are made faster, more efficient, and cost-effective through automation may be a great incentive to propel the development of solvent-free assembly methods. Although solvent-free colloidal assemblies may offer these potential advantages, this field remains in its infancy. This is elucidated by only one review about solvent-free assembly in the literature compared to the vast reviews on wet assembly. On the other hand, the assembly of larger granular beads has been extensively studied within the granular community for decades, including the electrostatic self-assembly of (sub)­mm-scale beads, as these dynamics are more dominated by gravitational and/or Coulombic interaction than by surface interactions present in dry colloidal systems.

Although the first reported dry assembly method of colloidal particles in the form of manual rubbing using bare fingertips was introduced in 1972 by Iler who failed to perfectly arrange an ordered monolayer of 50-μm silica particles on glass substrates,^13^ it regained attention after 2009 due to the groundbreaking work of Khanh and Yoon,^14^ and the Jeong group.^15^ The latter developed an innovative approach to achieve the rapid formation of particle monolayers, made of silica or polymer particles, on elastomeric (mainly PDMS) surfaces spanning a few tens of millimeters within just 20 s in a completely dry environment. The dry rubbing process’s simplicity, versatility, scalability, and cleanliness appealed to scientists as a promising alternative to traditional wet assembly methods. To expand the potential application of dry-assembled colloidal monolayers in the analytical^16^ or colloidal lithography^17^ domain, it is highly desirable to assemble ordered colloids on nonelastomeric target substrates. In this regard, a team led by Sotthewes and Jimidar recently reported their work unraveling the physical phenomena involved in the dry rubbing assembly,^18^ demonstrating that triboelectric charging and contact mechanics are key aspects for attaining ordered monolayers on rigid substrates. Triboelectric charging or contact electrification is the interfacial process in which two surfaces exchange electrical charges when rubbed against each other or when they are contacted and separated.^4,19^

Next to the rubbing assembly process, Jimidar and coworkers explored other solvent-free assembly methods, e.g., vacuum-assisted techniques^20,21^ and powder agitation,^5^ comprising monodisperse silica and polymer microspheres with diameters of 3 to 10 μm.

This perspective reviews the significant advancements in dry particle assembly over the past decade, aiming to significantly advance the fundamental aspects of this research domain and facilitate more sustainable assembly processes in device fabrication. It first introduces the unique advantages of dry assembly, particularly its rapid and straightforward approach to producing two-dimensional colloidal monolayers. The resulting structures are then compared to those produced by wet-assembly techniques. Finally, the current challenges and fundamental knowledge gap in the field are discussed, which may inspire the broader materials and interface science community to transform the solvent-free rubbing method from a promising alternative into a fully mature and widely adopted assembly technique.

## 2 The Advantages of Dry Particle Assembly

To showcase the promising nature of assembly without solvents, we discuss the topics in which the dry assembly method can compete with (or even surpass) wet assembly techniques in creating ordered particle monolayers. As such, different assembly aspects are compared in terms of ease of preparation and, of course, the quality of the final assembled structure. As the rubbing method has been predominantly used in the dry assembly domain, a major part of the assembly aspects is elaborated in light of the rubbing method. In some relevant instances, other dry assembly approaches are highlighted and compared to their wet counterparts, which we concisely describe, as many excellent reviews^10,22–24^ point out wet assembly in great detail.

### 2.1 Highly Ordered Monolayers of Spherical Particles

By rubbing dry powder sandwiched between two soft elastomeric substrates (*Y* ≈ 3 MPa), Park et al.^15^ achieved a perfect particle monolayer of silica and polymer colloids on a large scale (tens of mm). The radii of the colloids ranged from 100 nm to 5 μm. By sandwiching dry powder between two surfaces, pressure is applied to the particles, and a transferable momentum is attempted to induce movement in the particles. The top surface is called the stamp, while the bottom substrate is called the host substrate in this dry rubbing assembly process. To make a close-packed monolayer, Park et al.^15^ postulated that a rolling motion should be induced in the particles. Sliding particles may disturb the already close-packed ordering. Therefore, a certain force is needed to create a particle monolayer. Park et al.^15^ posed the following condition that needs to be met to create a monolayer:

1 $$\mu_{r}=\frac{F_{\mathrm{shear}}}{F_{\mathrm{p-s}}+P}\approx 1$$

with μ_r_ as the rolling friction coefficient, which, if approximately equal to 1, leads to rolling motion; *F*_shear_ as the shear force; *P* as the applied pressure during rubbing; and *F*_p–s_ as the particle–substrate interaction. The latter can be affected in three ways: (i) contact mechanics force between the particle and the host substrate, (ii) the applied force on the stamp (which also affects *P*), and (iii) triboelectric-induced attraction between the particle and the host substrate.^18^ When the particle–particle attractive energy is lower than the particle–substrate attractive energy and the particles can roll, a monolayer can be formed on the substrate.

The significant influence of the contact mechanics force alone explains why particle monolayers could not be achieved on rigid materials such as glass and SiO_2_ by using the dry rubbing method. However, when softer elastomeric materials were employed, the successful formation of particle monolayers was observed.^15,25–27^ If the host substrate or stamp is excessively sticky, the arrangement of the particles is compromised, as the particles lose their ability to roll freely across the surface. Controlling the applied force (or pressure) on the stamp is crucial to facilitate a rolling motion and prevent sliding.^15^ This applied pressure is highly sensitive; for instance, the optimal pressure required for 170 nm particles was eight times higher than that for 5 μm particles. Note that as particles become smaller, the interparticle interactions also become relatively stronger, leading to aggregation, which may partially explain an 8-fold increase in the applied pressure to separate the aggregates into single particles needed for the ordered monolayer assembly. While the rubbing speed also plays a role in the process, its effect is relatively minor. Thus, by carefully tuning the adhesion force and rubbing pressure, it is possible to consistently achieve particle monolayers on soft elastomeric surfaces consistently. It should be noted that Khanh and Yoon^14^ assembled a monolayer of 20-nm silica colloids on a polyethylenimine (PEI) layer covering a glass plate using the rubbing method. Although they could assemble a monolayer, it comprised more vacancies and grain boundaries compared to the larger particles they used, underscoring the challenges in assembling nanoparticles with relatively strong cohesive interactions.

While elastomeric substrates can be employed in applications that require flexible surfaces, other applications, such as (bio)­analytical or colloidal lithography, require more robust and inert target substrates.^3,17^ As such, a recent study by Sotthewes et al.^18^ addressed the research gap in understanding the physical phenomena occurring between the particles and substrates during the rubbing dry assembly by exploiting nonelastomeric substrates, opening avenues for other applications. They successfully attained monolayers comprising silica or polymer colloids (500 nm −10 μm) on rigid, nonelastomeric substrates with elastic moduli >20 GPa, e.g., Au-coated and SiO_2_-covered silicon wafers. It was found that particles with a lower stiffness (*Y* ≈ 5 GPa) were needed for conducting surfaces to attain ordered monolayers, while for insulating substrates, the triboelectric charging of the substrates improved the order of the assembled structures. As a result of the triboelectric effect, charge transfer occurs between two surfaces in contact during the rubbing process,^4,28^ adding an electrostatic attraction and, concomitantly, an adhesion component between the particles and the substrate. Thus, triboelectric charging becomes the dominant mechanism for achieving particle monolayer formation in cases where the substrate’s elastic modulus is high and the contribution from contact mechanics is minimal. The most effective results were achieved on CF_*x*_-coated surfaces, attributed to their relatively low elastic modulus (*Y* ≈ 21 GPa) and strong triboelectric properties.^29^ They corroborated their findings that the contact mechanics force and triboelectric charging are key contributors to obtaining sufficient adhesion between the dry powder particles and underlying substrates by utilizing atomic force microscopy (AFM) techniques, which included colloidal probe^29^ and Kelvin probe force microscopy (KPFM) measurements.^18^

Some results of ordered monolayers attained by using the dry rubbing method and various wet assembling methods are shown in Figure 1. With both methods, highly ordered, hexagonally packed monolayers can be created in which there is no difference observed in the general ordering. However, the dry method is easier, faster, and cleaner compared to the wet method, in which the solvent needs to be removed from the sample. The particle monolayers are created within 20 s and can be immediately used for postprocessing in applications. In addition, when the dry method is used, the long-range ordering for polymer-based particles is slightly better. Fewer domain boundary defects and larger grains are observed. This is especially observed when the rubbing procedure is unidirectional.^15,31,33,34^

**Figure 1:**
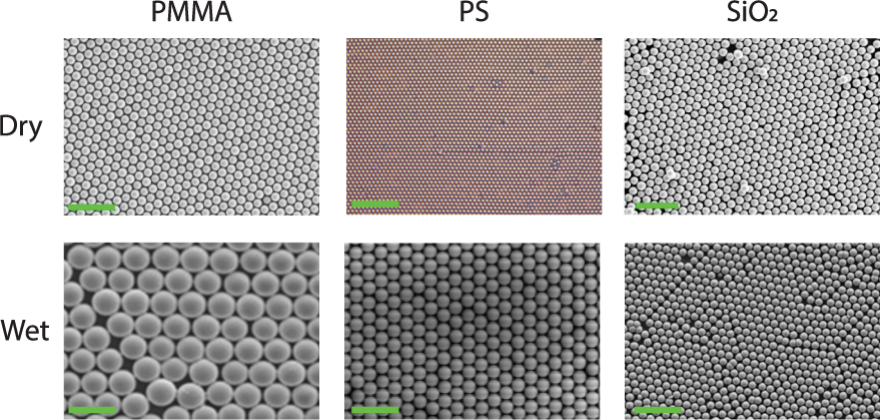
Overview of ordered colloidal monolayers created with the dry and wet method. Dry-PMMA: 10 μm PMMA on CF_*x*_ using the dry rubbing method. Reproduced with permission from ref. 18. Copyright 2024 American Chemical Society. Wet-PMMA: 3.81 μm PMMA on glass using drop-casting, scale bar = 7 μm. Reproduced with permission from ref. 30. Copyright 2004 American Chemical Society. Dry-PS: 1 μm PS on PDMS by using the dry rubbing method. Reproduced with permission from ref. 15. Copyright 2014 Wiley. Wet-PS: 4 μm PS on polyethylene by draining the water, scale bar 10 μm. Reproduced with permission from ref. 31. Copyright 2019 American Chemical Society. Dry-SiO_2_: 10 μm SiO_2_ on CF_*x*_ using the dry rubbing method. Reproduced with permission from ref. 18. Copyright 2024 American Chemical Society. Wet-SiO_2_: 2 μm SiO_2_ on glass by dip coating; scale bar 10 μm. Reproduced with permission from ref. 32. Copyright 2010 Sciendo.

For hydrophilic particles, comparable results are obtained using dry and wet methods (Figure 1, SiO_2_ column). In wet assembly, the solution can be tailored to match the wettability of the particles, allowing for the formation of monolayers with both hydrophobic and hydrophilic particles.^35^ In contrast, the quality of monolayers formed via the dry method is significantly influenced by the relative humidity of the surrounding air when dealing with hydrophilic particles. For example, due to the hydrophilic nature of SiO_2_ particles, capillary bridges tend to form between them, leading to aggregation. Although higher humidity enhances triboelectric charging, the increased particle–particle adhesion prevents the successful formation of monolayers.^29^ However, when experiments are conducted under low-humidity conditions (e.g., zero-humidity glovebox conditions), high-quality monolayers of hydrophilic particles can be obtained, comparable to those achieved in wet environments (see Figure 1).^18,36,37^

While most studies nowadays utilize “monodisperse” particles (low size distribution <2%) as chemical synthesis processes have been improved, polydispersity of the particles can influence the packing quality of the formed monolayer. In a wet environment, polydispersity below 9% has little impact on the crystalline structure and the number of defects and grain boundaries, and is essentially indistinguishable from that of a monodisperse distribution.^38–41^ On the other hand, above 15% polydispersity, no local minima exist corresponding to homogeneous (partially) ordered structures; therefore, the structure looks disordered.^42^ For the dry method, experimental evidence about the influence of polydispersity on the ordering is lacking, and a theoretical framework is missing. Recently, it was found that the formation of colloidal assemblies made with the dry-rubbing method is described by the (two-dimensional) critical nucleation theory (CNT).^37^ The CNT uses two terms to obtain an expression for the free energy of the formation of a nucleus. Particles at the perimeter add a positive energy term through the edge line tension, and particles in the bulk add a negative term due to the free energy gained from bonding to a crystal. A similar theory was used to explain the formation of colloidal assemblies in a wet environment.^38^ Auer and Frenkel^38^ found that a polydispersity higher than 9% influences the assembly outcome, and more defects/grain boundaries occur. Due to the similarities between the two theories, a similar result might be expected for dry conditions, but experimental evidence or numerical modeling does not (yet) exist to confirm this.

In short, both dry and wet methods effectively produce large-scale, highly ordered particle monolayers with particle sizes ranging from 100 nm to 10 μm. Nevertheless, dry assembly approaches have proven to offer several advantages compared to wet methods: they can be significantly faster (<20 s vs hours for wet assembly), more straightforward (requiring only two surfaces and the particles) without requiring highly optimized conditions (e.g., temperature, pH, evaporation rate), scalable and applicable for a broad range of particle sizes, and cleaner (eliminating the need for solvents while eradicating stains and solvent contamination). In addition, even on a ∼cm scale, dry-assembled monolayers may only have a few grain boundaries and excess particles on top as a defect, whereas with wet methods, such defects are more common and conspicuously present, which can limit the performance of devices, e.g., antireflective surfaces.^17^ Furthermore, the dry rubbing method is more amenable to automation, rendering the assembly method more efficient. Such automation processes have recently been demonstrated between two PDMS surfaces by ten Napel et al.,^43^ Park et al.,^44^ and Tzadka et al.^17^ for high-throughput nanofabrication.

### 2.2 Confined Particle Monolayers of Spherical Particles

In contrast to large-scale monolayer microparticle assembly, ordered monolayers in specific array geometries or superstructures can also be created for applications in colloidal lithography, bioanalytics, or microengineering of parts of devices with a specific functionality. The assembly of such patterned monolayer arrays can be broadly divided into two categories: (i) physical and (ii) chemical templates. Physical templates are based on solid substrates with prestructured cavities of the desired size and shape. These structures are often produced by using a lithographic technique. Chemical template pattern substrates are prepared using surface chemistry in combination with nano/microstructuring processes, resulting in regions with higher and lower affinities for the colloids to be located.

#### 2.2.1 Physical Templated Surfaces

The self-assembly of 2D crystals on surfaces devoid of structures typically results in a crystal lattice with the lowest free energy, most often a hexagonal arrangement (Figure 1 and Section 2.1). Therefore, surfaces containing predesigned spaces, cavities, or interfaces acting as traps or nucleation sites (depending on their size) are utilized for particles^22^ to attain the desired lattice structure. Such physical structures can be obtained by using lithography techniques combined with wet or dry etching techniques in either photoresist films, silicon wafer surfaces, or any other substrates.

Particle assembly on physically templated substrates is commonly employed in both dry and wet assembly methods. Using predesigned templates, other packing geometries can be obtained, such as square packing, as shown in Figure 2a,b. In a wet environment, a drying front was used (Figure 2b) to create the monolayer, in which a colloidal suspension is injected between two plates from which one is patterned with grooves.^45^ The liquid evaporates, driving a drying front over the plate and depositing the particles in the grooves. Similar to other wet assembly approaches, this process is highly dependent on the particles used (size and material properties), solvent, evaporation rate, and substrate. Khanh and Yoon^14^ performed the same experiment without solvents using the rubbing method (Figure 2a)^14^ on a patterned silicon substrate covered with a sacrificial polymer layer. Similar results are obtained, but the process is faster, simpler (independent of highly optimized conditions), and cleaner. In addition, cracking due to particle shrinkage is avoided, and the process can easily be scaled up to wafer scale.

**Figure 2:**
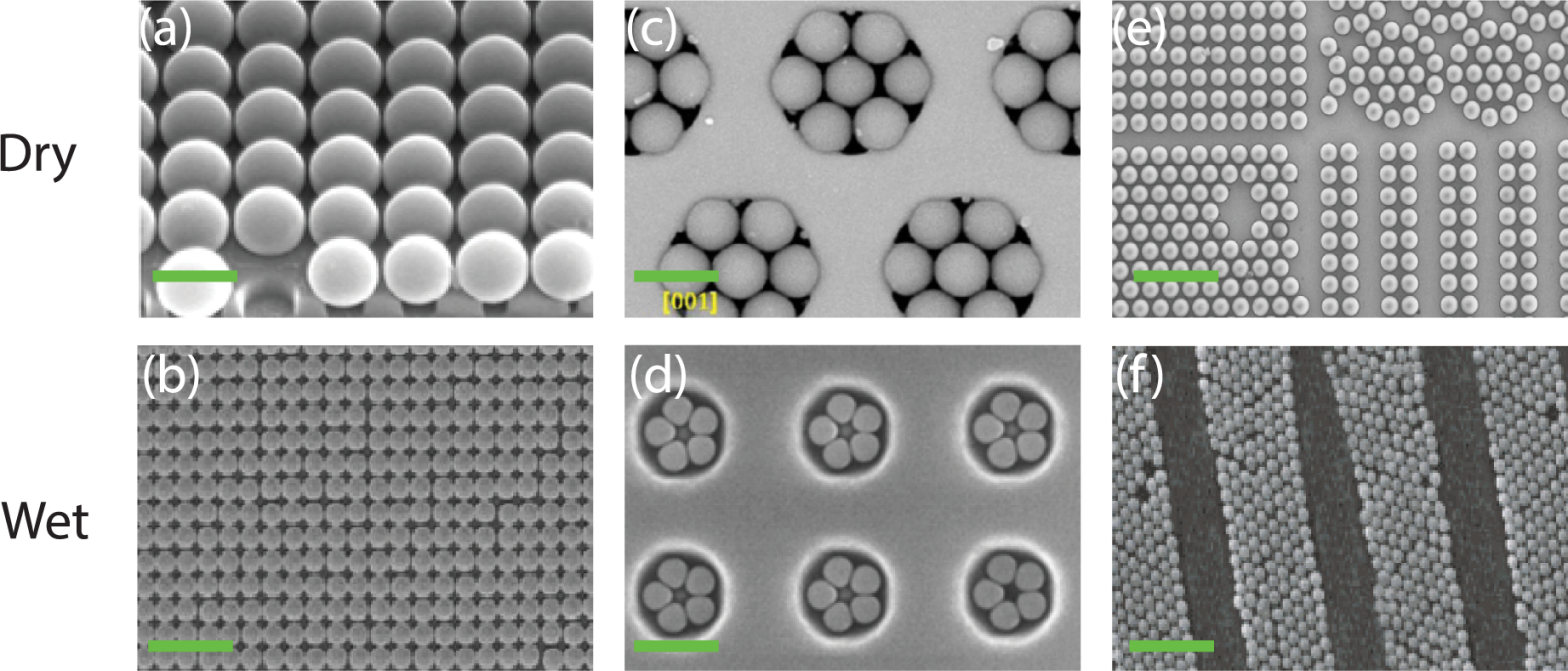
(a) 0.7 μm silica particles on etched Si wafers using the dry rubbing method; scale bar, 0.7 μm. Reproduced with permission from ref. 14. Copyright 2009 American Chemical Society. (b) 1 μm polystyrene particles on a polycarbonate grooved surface using the drying front technique; scale bar 4 μm. Reproduced with permission from ref. 45. Copyright 2008 Wiley. (c) 3 μm polystyrene particles in hexagonal holes made in PMDS using dry rubbing method, scale bar 4 μm. Reproduced with permission from ref. 46. Copyright 2016 American Chemical Society. (d) 0.7 μm polystyrene particles in pentagon edged Si using attractive capillary forces. Reproduced with permission from ref. 47. Copyright 2001 American Chemical Society. (e) 10 μm polystyrene microparticles using vacuum-driven assembly; scale bar 45 μm. Reproduced with permission from ref. 20. Copyright 2022 Elsevier. (f) 0.58 μm PMMA particles on holographic gratings in photoresist using electrodeposition, scale bar 4 μm. Reproduced with permission from ref. 48. Copyright 2002 Wiley.

The sacrificial polymer in the work by Khanh and Yoon^14^ was needed to promote contact mechanics force, i.e., adhesion, between the particles and the cavities. After assembly, the polymer layer was removed at 500 °C, limiting the use of assembling polymer particles with lower melting temperatures. The dry rubbing assembly without a sacrificial polymer layer was later attempted for silica and polystyrene particles by Verloy et al.,^16^ but it resulted in many vacant wells on a silicon surface. The confinement force of the wells was too low to compensate for the loss of the adhesion force of the adhesive layer. However, they adapted a hybrid wet–dry assembly process by introducing a wet rubbing assembly process, i.e., a particle suspension rubbed across the silicon-templated substrate using PDMS and were successful in attaining an ordered array for a broad spectrum of particle types and sizes dispersed in water, ethanol, or IPA, highlighting the ease and simplicity of the rubbing method.

Systematic variation of the size of wells on a surface has also been shown to lead to the defined assembly of colloidal clusters with an adjustable number, determined by the ratio of diameters between surface structures and colloids.^14,46,47^ A high degree of order can only be achieved when the size of the cavities is commensurate with a distinct number of colloids. Yin et al. first attained this using template-assisted wet self-assembly of polystyrene particles on patterned Si (Figure 2d).^47^ Similar to the drying-front technique, the liquid was slowly removed from the parallel plate configuration, forcing the liquid front to move and deposit the particles in the cavities. On the other hand, a comparable result was obtained much faster with the dry rubbing method.^14,46^ Another significant advantage is the pressure applied to the particles during the rubbing process, which forces the particles to enter already occupied cavities, leading to closer packing densities, particularly in combination with cavities on an elastomeric substrate (Figure 2c,d). The assembly of such ordered structures inside a cavity can be readily scaled up to a much larger scale, e.g., a full wafer, using the dry rubbing method.^46^

Besides the dry rubbing method and evaporative front wet assembly method, electric field-assisted methods can also achieve ordering on predesigned surfaces. Two examples are shown in Figure 2e,f. In the dry case (Figure 2e), vacuum-driven assembly is used, which is based on electric field-assisted fluidization of dry powder into single particles.^49^ Instead of cavities, the predesigned wells are perforated to allow a strong vacuum pressure to attract the single particles to their positions in the electrostatic cell. In this way, every predetermined pattern can be made of various types of particles,^20^ leading to the assembly of more than 160,000 silica microspheres, each of 5 μm in diameter, precisely on a prestructured silicon grid in the order of a few seconds (≈ 8 s). Another advantage of the vacuum pores was that particles could be transferred onto another substrate, and the physically templated materials were recycled for the next assembly process, adding to the sustainability aspects of the dry assembly. Furthermore, this vacuum-assisted assembly process is amenable to automation, paving the way for a time-efficient assembly. In a wet environment (Figure 2(f)), electrodeposition can be used, which entails a particle suspension placed between two plates with a voltage applied across them.^48^ The particles are moved toward the desired location using electrophoresis. The exact ordering of the particles heavily depends on the commensurability between the particles and the desired geometry, with some apparent defects.

In summary, both wet and dry methods can be utilized to assemble any confined particle monolayer configuration on physically patterned surfaces. It can be inferred that the dry methods, e.g., rubbing or vacuum-assisted assembly, are faster and have a higher tolerance to achieve a higher packing density in a controlled manner compared to the wet assembly methods, underscoring the potential of solvent-free assembly methods. In addition, designing and fabricating physically templated substrates that can be recycled for multiple assembly processes may add value in establishing more sustainable approaches, particularly for solvent-free assembly processes.

#### 2.2.2 Chemically Templated Surfaces

An inexpensive method for a pre-engineering substrate that has proven to be equally effective as physical templating is by means of chemical surface functionalization, i.e., chemically patterned surfaces. The predesigned areas with distinct chemical functionalities allow for site-selective particle attachment by modifying properties such as adhesion.

The first class of patterns is based on a difference in adhesion caused by electrostatic attraction/repulsion changing particle adhesion. Such pattern functionality can be realized using microcontact printing, among others. Figure 3b shows the result of particles that were selectively assembled on microprinted PMA-*co*-PDDA monolayers on Si.^50^ With microcontact printing, patterns of various shapes can be created. Using the droplet evaporation method, a 3D colloidal crystal formed over the entire surface. Subsequently, the surface was rinsed repeatedly with deionized water and dried with nitrogen gas. Only the particles that are located on the PMA-*co*-PDDA remain on the surface due to the higher adhesion. A similar approach is used in dry environments by Sotthewes et al.^18^ They covered a Si wafer with CF_*x*_ patterns using lithography and plasma polymerization. By rubbing over the surface, the particles preferentially stuck to the CF_*x*_ layer instead of the Si surface (Figure 3a).^36^ The adhesion of the CF_*x*_ layer is so much higher because of the triboelectric-induced charges between the layer and the particles during the rubbing process and the high contact mechanics force. Using this technique, any desired shape can be manufactured on a wafer scale (Figure 3c,e). The method allows us to investigate the nucleation of the particles and the influence of the pattern geometry on the final ordering of the monolayer.^37^ So far, this approach has only been limited to a particle size of 3 μm and remains an unexplored avenue for smaller particle sizes. The challenge for smaller particle sizes is the relatively higher adhesion forces, which may hinder the selective assembly of smaller beads on the CF_*x*_ coating. As such, an in-depth fundamental understanding of all surface interactions present is needed to advance this assembly approach to even smaller particles.

**Figure 3:**
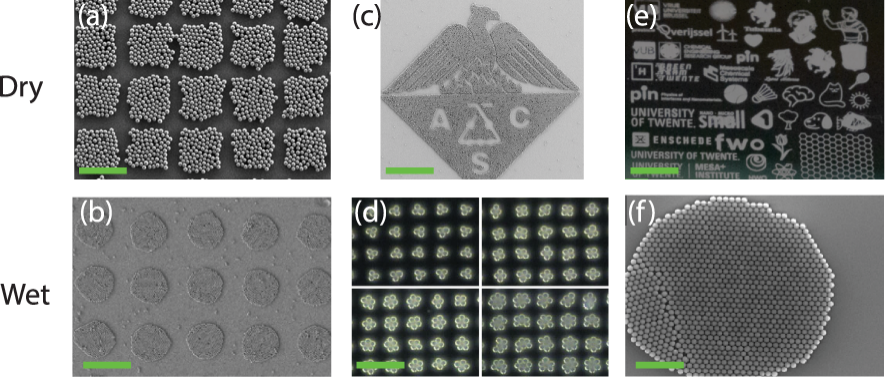
(a) 5.0 μm silica particles on lithography CF_*x*_ patterned Si wafers using the dry rubbing method; scale bar 50 μm. Reproduced with permission from ref. 36. Copyright 2020 American Chemical Society. (b) 0.4 μm polystyrene particles on a microcontact printed PMA-*co*-PDDA patterned Si wafer by immersion in a particle solution; scale bar 12 μm. Reproduced with permission from ref. 50. Copyright 2010 American Chemical Society. (c) 3 μm PMMA particles on lithography CF_*x*_ patterned SiO_2_ wafer using the dry rubbing method; scale bar 40 μm. Reproduced with permission from ref. 18. Copyright 2024 American Chemical Society. (d) 4.30 μm carboxylated polystyrene particles on a microcontact printed polyelectrolyte patterned Au surface using the solution evaporation method, scale bar 40 μm. Reproduced with permission from ref. 51. Copyright 2002 Wiley. (e) 3 μm PMMA microparticles on lithography CF_*x*_ patterned 4-in. Si wafer using the dry rubbing method, scale bar = 4 mm. Reproduced with permission from ref. 18. Copyright 2024 American Chemical Society. (f) 1 μm SiO_2_ particles on a self-assembled octadecyltrichlorosilane on Si using the droplet evaporation technique; scale bar 10 μm. Reproduced with permission from ref. 52. Copyright 2005 American Chemical Society.

Small clusters of particles can also be created on microprinted patterns after the substrate is placed in a suspension. For example, polyelectrolyte patterns are made on a Au-coated surface, and particles tend to stick to these patterns due to electrostatic interactions after rinsing off the main solution (Figure 3d).^51^ The technique offers a high level of control, allowing for the formation of clusters as small as three particles, although with a reduced yield. The shape and size of the particle clusters are determined by the ratio between the pattern diameter and the particle diameter. Currently, the smallest number of precisely arranged particles achieved using a dry rubbing method on a chemically patterned substrate is around 10.^37^ Further advancements are required to achieve the same level of selectivity as wet methods for creating single-particle patterns, i.e., accurate positioning of an individual particle on chemically templated substrates.

Another method to create confined particle layers using chemical template assembly is changing the wettability of the surface (Figure 3f).^52^ Changing the surface wettability of the patterns makes the droplet (solvent) sensitive to the surface, leading to the assembly of particles within the droplet on the preferred surface of the droplet. A special case of using wettability to align particles is with the alignment of Janus particles.^53^ The Janus particles consist of a polystyrene particle that is half coated with Au and exhibits random orientation on a neutral substrate. When immersed in water, the orientation of the Janus particles on the surface depends on the surface wettability as a result of the arrangement of the water molecules. Due to the differing wettability, the local water density near the surface varies, leading to a different orientation of the Janus particle.

In dry environments, wettability is less suitable for the action of a separation agent. Although the adhesion between a hydrophilic particle and a hydrophilic surface increases with increasing relative humidity,^29^ particle–particle adhesion also increases, thereby impeding particle selectivity. As such, until now, the alignment and assembly of Janus particles using dry assembly methods have not been broadly explored, but the Jeong group reported a study that assembled Janus particles comprising hemispherical parts of two different sizes using the rubbing method (Section 4). Giving Janus particles an anisotropic magnetic^54^ or metal-dielectric^55,56^ property may help exploit their alignment in dry assembly, as demonstrated before in wet environments.

Thus, covering a substrate with patterns of different chemical functionality than the host substrate is a less expensive strategy to attain ordered monolayer arrays of particles. The chemical pattern can selectively increase the interaction between the pattern surface and the particles. At the same time, chemical patterns may also affect the wettability, which has direct selectivity toward the droplet comprising the particles. The latter is inapplicable for solvent-free assembly methods, whereas the tribocharging-induced selective adhesion of particles on CF_*x*_-patterned substrates results in ordered assembly of a broad spectrum of particles rapidly. However, when creating small particle clusters, wet methods are more reproducible in attaining the desired result, emphasizing the need to understand the interaction forces at the micro- and nanoscale in dry assembly.

#### 2.2.3 External Stimuli Assembly

When particles are submerged in a solution, they float in the solution and are therefore susceptible to external stimuli, such as electric and magnetic fields, light, or the deposition method itself. For instance, illuminating indium tin oxide (ITO) through a mask with UV light results in a small increase in current density in the material. A pattern of high and low current densities is generated across the surface. As a consequence of electro-osmotic and electrohydrodynamic flows, induced by the difference in current density, the particles are confined and form the desired pattern.^57^

Magnetic fields can also help arrange particles, although not as effectively as the above-described methods. Using an external magnetic field, Janus particles are aligned along the applied field.^58^ For more examples, the reader is referred to ref. 59.

So far, external stimuli have been successfully applied for the solvent-free arrangement of beads down to 40 μm^60,61^ in which potential wells can trap particles by applying a voltage. For an elaborate overview of dry assembly methods applying external stimuli to attain ordered structures, the interested reader is kindly referred to the recent review.^3^ However, for smaller particles (<10 μm), this is even more challenging due to the much stronger surface forces, as was attempted by Jimidar et al. on a CF_*x*_ patterned Si substrates under the influence of a strong electric field.^49^ Although they were able to selectively capture particles in the CF_*x*_ patterns, the order of the monolayer was poor, highlighting both the potential and the challenges involved in the solvent-free assembly of a colloidal powder. Such challenges in the ordering of microparticles can be resolved by using physically templated surfaces, as discussed in Section 2.2.1 (Figure 2e), whereas for nanoparticles, one may first need to disintegrate aggregated particles by mechanical impact, for example, before using an electric field to assemble single particles in an ordered monolayer. Thus, the electric field alone will possibly not be sufficient to overcome the cohesive interactions in the dry nanoparticle powder, making it a less efficient method.

## 3 Challenges and Opportunities for Dry Particle Assembly

Up to this point, our concise overview has underlined that dry assembly methods can compete or even exceed wet assembly methods in terms of assembly time, scalability, versatility, scarce defects, and automation to attain ordered monolayers comprising monodisperse spherical colloids. However, what follows are assembly avenues that have been almost solely related to the wet assembly domain. As such, some of these wet assembly techniques are explained, and the challenges for dry particle assembly methods are identified.

### 3.1 Highly Ordered Particle Multilayers

Expanding colloidal monolayers to an extended dimension in space and complexity leads to three-dimensional structures. This domain is dominated by wet assembly techniques, which provide the opportunity to make multilayer-ordered colloidal assemblies as well as more advanced structures such as supraparticles through spherical confinement (self-assembly within droplets).^41,67^ Here, we will only discuss the formation of multilayers on solid substrates as, so far, no solvent-free methods exist that can assemble multilayers of colloids floating in air, i.e., not on a substrate. For more information about the formation of 3D colloidal crystals, the reader is referred to the following refs 22,23 , and^41^.

We point out that our discussion is focused on assembled structures using colloids with a low size distribution as syntheses have improved. However, as defects may still occur during chemical synthesis, e.g., secondary nucleation and polydisperse samples may still exist experimentally. It is reported that a particle size distribution of ≲5% has little effect on the phase diagram and packing structures. In addition, the particle “hardness″ seemingly affects the crystallization process compared to a monodisperse case; as for PMMA, an ≈5% distribution is sufficient, whereas polystyrene requires a sharper distribution (≈1%). For a more elaborate discussion on the effect of particle polydispersity and 3D crystallization, we refer the interested reader to a recent review by Royall et al.^41^

In the beginning, the growth of 3D colloidal crystals was achieved through sedimentation, which relies on the combination of gravitational force, Brownian motion, and crystallization. The produced crystals contain fcc lattices, which are the most favorable orientation, and a random number of layers. An example is shown in Figure 4a, which illustrates the result of placing a PMMA slab in a SiO_2_ particle-containing solution for 2 to 4 weeks.^62^ The resulting lattice structure depends on the sphere diameter, and the number of layers present is random. The assembly process is determined by balancing the sedimentation rate and thermal energy such that the largest assembled particle using this method is 0.5 μm.

**Figure 4:**
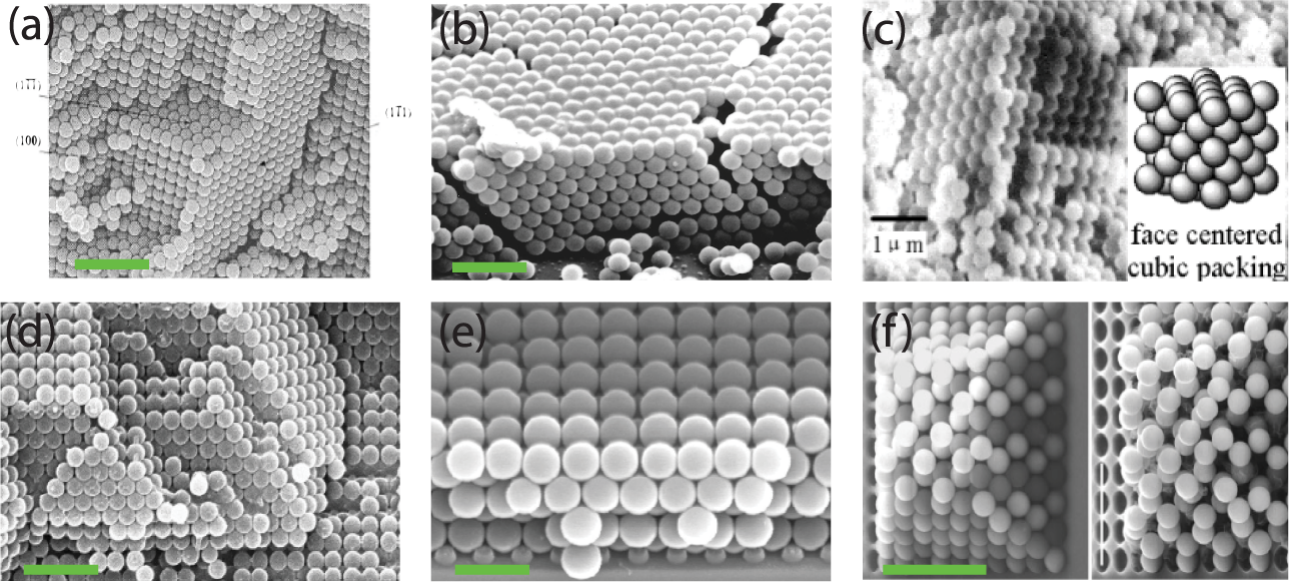
(a) 0.4 μm SiO_2_ particles on PMMA using the sedimentation technique. This is the result after 2 to 4 weeks, scale bar 3 μm. Reproduced with permission from ref. 62. Copyright 1997 American Chemical Society. (b) 0.4 μm SiO_2_ particles on a glass substrate using dip coating; scale bar 2 μm. Reproduced with permission from ref. 63. Copyright 1999 American Chemical Society. (c) 0.4 μm P­(St-MMA-AA) colloidal latex particles on a textured Si wafer with the crystal fcc packing, scale bar 1 μm. Reproduced with permission from ref. 64. Copyright 2003 American Chemical Society. (d) 0.3 μm polystyrene particles in a (100) oriented crystal grown by template-assisted colloid assembly, scale bar 1.5 μm. Reproduced with permission from ref. 65. Copyright 2005 American Chemical Society. (e) 0.7 μm SiO_2_ particles on a square patterned Si wafer using the dry rubbing technique, scale bar 1.5 μm. Reproduced with permission from ref. 14. Copyright 2009 American Chemical Society. (f) Six-layer structure of 1.2 μm latex and silica particles before (left, BCC structure assembled using nanorobotic pick-and-place setup) and after (right, diamond structure) plasma etching, removing the latex particles, scale bar 5 μm. Reproduced with permission from ref. 66. Copyright 2002 Wiley.

In the vertical deposition (dip coating) technique driven by evaporation, the capillary force is the driving mechanism behind the assembly of SiO_2_ particles presented in Figure 4b. The host substrate is repeatedly immersed into a particle solution at an inclined angle with drying steps in between of at least 24 h.^63^ Similar to sedimentation, only fcc structures can be created, but in this case, the number of layers can be controlled by the particle diameter and particle volume fraction. The obtained colloidal crystals exhibit defects, cracks, and grain boundaries, which are caused by drying tension. Using the same technique, but now in a viscous solution comprising latex colloidal particles, it is possible to create fcc and hcp colloidal crystals (Figure 4c). These distinct observations exemplify that the ordered lattice structure depends on the particle type in wet assembly. Furthermore, as is typically the case in wet assembly, the temperature and surface tension of the solution can be altered to tune the crystal orientation.

The methods described above showcase the efficacy of assembling ordered multilayers, albeit very slow, using wet assembly. The assembly of such multilayer structures can be faster using other techniques that include spin-coating, doctor blade, the coffee stain effect in sessile droplets, filtration, and microfluidic cells. However, all of these techniques rely on principles that cannot be mimicked by dry assembly methods, as a liquid medium is absent to tune sedimentation, Brownian motion, and capillary forces. The latter has proven to be too weak to order particles without solvents. If anything, capillary forces have mainly been a bottleneck when assembling monolayers from dry powder, as shown in previous studies using the rubbing method.^3,18^

These challenges explain why almost all 3D colloidal structures have been produced by using wet assembly techniques. There are methods to levitate particles in a dry environment, for instance, using electric fields^49^ or acoustic levitation.^68^ To let small particles (<10 μm) levitate, the driving force needs to overcome dominating surface adhesion forces, which are more difficult to overcome and control than those for larger beads. As such, levitation using electric fields is used to assemble particles on chemically and physically templated surfaces; however, no multilayer particle ordering was found, but resulted in only randomly aggregated particle clusters.^49^ However, using a multifaceted approach to disperse dry powder in air, followed by a subsequent controlled capture (or sedimentation) of the beads for assembling 3D-ordered structures, may unlock the potential for dry colloidal assembly methods.

Hierarchical structures with colloids can also be achieved using colloidal epitaxy or template-assisted growth.^65,69^ A patterned substrate is made with cavities that have commensurate spacing and predefined symmetry. Using this method, crystals with the fcc (100) plane orientation can also be assembled in contrast to the regular (111) crystallization on unpatterned substrates (Figure 4d). The first layer, determined by the predesigned cavity symmetry, dictates the formation of subsequent layers. The features of the lattice-dictating pattern share the same dimensions as the colloidal particles. This restriction limits the minimum particle sizes (high monodispersity required) that can be used in the assembly process, as they need to match the spatial resolution of photolithography. This can be solved by introducing trenches, e.g., V-shaped channels, that can be filled with particles of varying diameters, i.e., a polydisperse sample, which all align in the same manner inside the channels.^70^

The template-assisted growth of multilayer-ordered colloidal structures in a so-called skeleton structure in a wet environment shows many similarities with physically templated surface growth (Section 2.2.1), but with rationally deeper cavities designed to host more particles and create 3D structures. After assembling the 3D-ordered structures, the host sacrificial skeleton structures are usually removed using dissolution or etching, which may lead to contamination or damage to the particles. As dry assembly methods have proven successful in rapidly assembling 2D-ordered lattices on physically templated surfaces, their utilization to attain multilayer-ordered structures using template-assisted growth structures (permanent or sacrificial skeletons) with deeper cavities seems a promising avenue for pursuing multilayer structures.

Khanh and Yoon^14^ attempted to assemble a multilayer of ordered structure using the dry rubbing method on a 2D-physically templated silicon substrate covered with a sacrificial polymer coating. After assembling a first anchoring layer of 0.7 μm SiO_2_ particles on the templated-silicon substrate, they assembled a silica particle multilayer, albeit with a maximum of 5 layers (Figure 4e), on the structured substrate using the solvent-free rubbing method. The multilayer assembly of same-sized silica beads on a larger scale is challenging, which can be ascribed to the positional instability of the particles in the layers higher than the first layer, in conjunction with the smaller gap sizes created by the four neighboring silica beads.

As already discussed, a rolling motion is required to create ordered colloidal structures by using the rubbing method, which also holds for 3D-ordered structures. As discussed in Section 2.1, eq 1 needs to be satisfied to attain a particle monolayer.^15,18^ When a second layer needs to be formed, the parameters of eq 1 drastically change; the particle–substrate interaction is replaced by the particle–particle interaction, and the shear force also changes. In addition, inevitably, the first particle layer represents (in the ideal case) a periodic curved surface instead of a flat substrate. This implies that the changing parameters will heavily affect the required pressure (and velocity, to a lesser extent).

Obtaining more information on the exact parameters during the rubbing process is necessary to estimate the required pressure in eq 1. This is no easy task, as the components of the particle–particle interaction (the contact mechanic force, including the van der Waals force), the electrostatic force, and the capillary force are heavily dependent on each other, which means that the exact conditions need to be precisely controlled.^3^ A preliminary demonstration by Khanh and Yoon^14^ showed that when the respective conditions are fulfilled, they could at most assemble five layers, albeit on a relatively small scale.

On the other hand, a robotic study^66^ explored the pick-and-place concept to produce a multilayer-ordered crystal structure comprising 1.2 μm silica and latex particles without solvents in a nanorobot setup. These particles were precisely placed on designated spots on a predesigned microstructured silicon surface (Figure 4f). In agreement with the rubbing study of Khanh and Yoon,^14^ the physically templated Si surface covered with a polymer adhesive ensured that the first layer was firmly anchored for the additional layers on top. The subsequent layers of particles are stacked in a fashion reminiscent of the arrangement of oranges in a market display. To demonstrate that open 3D-ordered structures consisting of silica particles alone can be attained, the PS particles were removed using oxygen plasma etching. Note that a polymer gluing layer was not needed on the silicon surface here because individual particles were meticulously positioned by the nanorobot compared to the vigorous rubbing method that could remove particles from the cavities during rubbing. By changing the size of the pickup needle, even small nanoparticles (<100 nm) can be arranged using the pick-and-place concept.^71^ The interested reader is referred to the recent work of Shah et al.^72^ for a more elaborate overview of robotic pick-and-place setups used in ordered colloidal assembly.

Instead of using a pick-and-place procedure, a push-and-scan procedure can also be followed.^73^ Here, an atomic force microscope is used to identify the location of the particles, and the tip pushes the particle to the correct position. Using this method, it is not possible to make 3D structure. However, by using a hole structure (physical template), particles are pushed inside the hole and an additional particle is pushed onto the top surface of the first particle layer, forming a 3D assembly.^74^ Unfortunately, the push-and-scan procedure is not capable of producing large, arranged crystals.

The pick-and-place nanorobotic strategy is appealing as a completely solvent-free technique, but it is time-consuming, taking ≈7 min to precisely position a single particle in the lattice. As such, this approach is infeasible to adopt, as its time efficiency is rather poor. While this is true, the robotics field, in particular the soft robotics field, has made significant advancements, offering promising prospects for faster particle pick-and-place procedures by using mechanical grippers with, for example, thermoresponsive polymer properties. The pick-and-place procedure can be accelerated if assembled ordered monolayers can be stacked in this manner with perfect alignment.

The two studies highlighted above used physically templated substrates to attain 3D-ordered structures. In contrast, on chemically templated surfaces (Section 2.2.2), second layers (or particles, as shown in Figure 1, dry-SiO_2_) are occasionally found, but the stability of this layer is much lower due to the lower adhesion forces. In general, the particle–particle interactions are much weaker than particle–substrate interactions, leading to instabilities in all the layers except the first one.^3^ As such, the adhesion between the particle and substrate, i.e., the first layer, on a chemically templated surface should be strong enough to sustain the rubbing motion if one pursues the rubbing method to assemble a multilayer-ordered crystal structure. Another path is to enhance particle–particle interactions. This can be achieved by covering the first layer of particles with a buffer layer, which affects the particle–particle interaction, or by functionalizing the particles after assembly. Unfortunately, this would add an extra step to the process, affecting the ease and rapidity of the method.

In conclusion, the assembly of 3D-ordered structures underscores that the solvent-free assembly field has yet to mature enough to compete with the wet assembly approaches in this regard. The presence of a particle-holding liquid medium enables the possibility of controlling particle deposition, which is not possible under dry conditions. In addition, the sufficiently strong surface interactions for smaller particles (<10 μm) under dry conditions make the application of assembling floating particles extremely challenging. On the other hand, it has been demonstrated that physically templated substrates may be advantageous to attain 3D-ordered crystals without solvents using the rubbing method or a nanorobotic pick-and-place setup. Overall, a deeper understanding of all the surface interactions at the micro- and nanoscale is needed to advance the solvent-free assembly field.

### 3.2 Binary Monolayers

Instead of monodisperse particle assemblies, binary (or ternary) mixtures of particles with different sizes, chemical nature, wettability, or combinations thereof can be used to create ordered particle layers. The combination of two particle types to prepare what are classified as binary colloidal monolayers in the literature adds to the complexity of the assembly approach and the number of parameters that need to be controlled to achieve satisfactory long-range order and stoichiometry. Such binary monolayers offer surface micro- and nanotopographies with heterogeneous chemistry and composition. Therefore, they can be applied in photonic devices, biomedical devices, cell culture substrates, biosensors, information displays, and optical imaging.^75,76^ A more detailed elaboration on the assembly of binary particle monolayers can be found in the following reviews:^10,22,75,77^ .

Binary monolayers of large and small colloids are generally made using two strategies: (i) from a mixed dispersion, i.e., coassembly or (ii) layer-by-layer assembly. Figure 5a shows an example of the first method, in which the large polystyrene particles form a closed-packed monolayer and the small polystyrene particles occupy the intermediate spaces.^78^ To ensure proper crystallization, exact control over the stoichiometry of the two particle populations at the interface is crucial.

**Figure 5:**
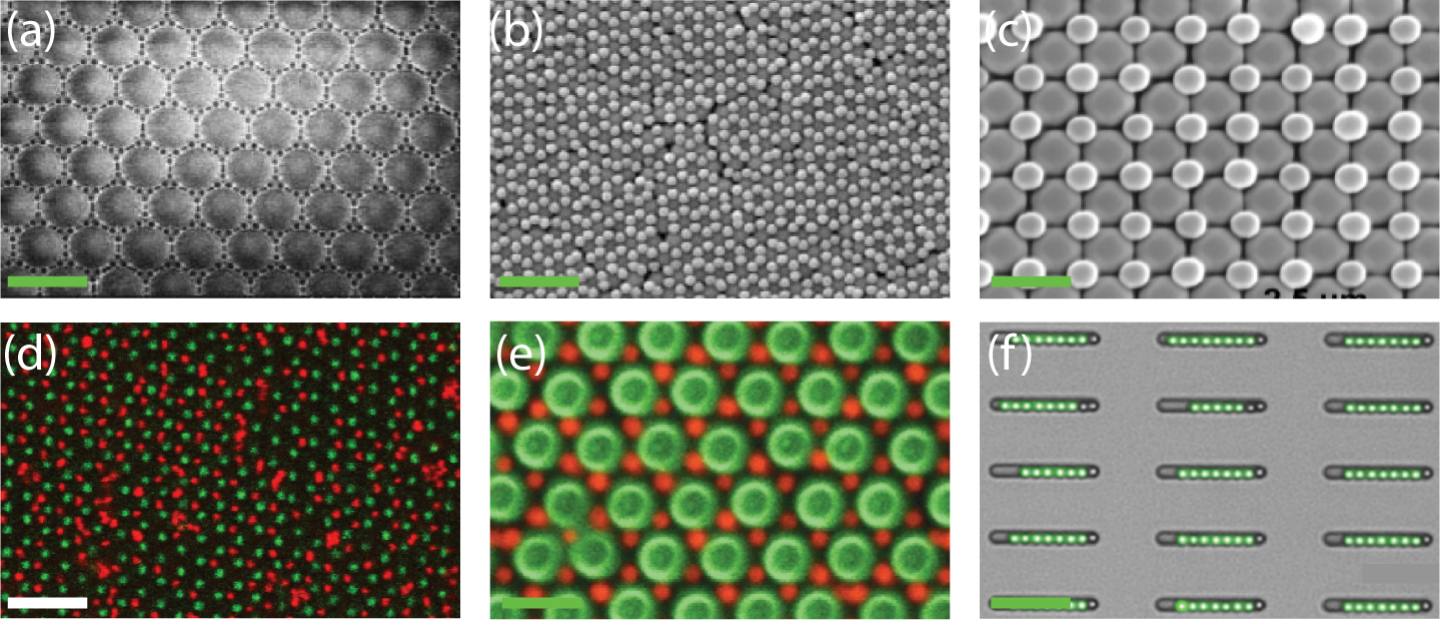
(a) Binary colloid assembly of large 1.28 μm and small 0.2 μm polystyrene particles using convective vertical deposition, scale bar 1.7 μm. Reproduced with permission from ref. 78. Copyright 2003 Wiley. (b) Binary colloid assembly of large 0.2 μm SiO_2_ and small 0.1 μm SiO_2_ particles using the controlled drying process on a vertical substrate, scale bar 2 μm. Reproduced with permission from ref. 79. Copyright 2003 Science. (c) Binary colloid assembly of 700 and 420 nm silica colloids using the dry rubbing method; scale bar = 2.5 μm. Reproduced with permission from ref. 14. Copyright 2009 American Chemical Society. (d) Cubic rock salt unit cell (111-plane) of 0.4 and 1.4 μm silica particles created by electric field-assisted crystallization; scale bar = 8 μm. Reproduced with permission from ref. 80. Copyright 2009 PNAS. (e) Binary crystal consisting of 1.5 μm paramagnetic (red) and 2.5 μm nonmagnetic (green) particles assembled on a hexagonal grid using magnetic molds, scale bar 5 μm. Reproduced with permission from ref. 81. Copyright 2013 Nature. (f) Colloidal chain of a 1 μm SiO_2_ head particle followed by a tail of 1 μm polystyrene particles, scale bar 10 μm. Reproduced with permission from ref. 82. Copyright 2016 Science.

The second strategy involves layer-by-layer deposition, which is a two-step process in which, first, a closed-packed monolayer of the larger constituents is crystallized on the surface, followed by the assembly of small particles. The interstitial sites of the large particle monolayer prove to be energetically favorable positions for the small particles to nucleate and form a superlattice (Figure 5b).^79^ The smaller particles are deposited by convective or electrophoretic assembly or electrostatic attraction.

By adjusting both the size and chemical properties of the particles, a wide variety of structures can be achieved using the layer-by-layer method.^83^ Modifying the size of the particles in the second layer allows fine-tuning of the optimal sphere positions. For example, open crystal structures can be created by assembling binary multilayers composed of polystyrene and silica particles. Upon heating the sample, the polystyrene particles melt, leaving behind the silica stacking (open structure), which forms hexagonal or rectangular arrays. While the resulting crystal no longer contains binary particles, it exhibits different crystal arrangements. This method of sequentially adding colloids or particles of varying sizes to form ordered layers can be repeated as required to achieve the desired number of layers.^84^ This process resembles the nanorobot pick-and-place setup^66,72^ used for the solvent-free assembly of an open crystal structure discussed in Section 3.1.

The coassembly as well as the layer-by-layer concepts are promising opportunities to explore in the dry assembly domain. Adapting the layer-by-layer approach to a dry environment presents challenges similar to those discussed in the context of the rubbing assembly in Section 3.1. Specifically, achieving the formation of a second particle layer requires overcoming significant changes in the parameters outlined in eq 1, which are highly dependent on the particle size and type. Despite these challenges, Khanh and Yoon^14^ exploited the layer-by-layer strategy to attain a binary monolayer of 0.7-μm silica particles and smaller silica particles (0.3 and 0.4 μm) in the interstitial spaces (Figure 5c). It should be noted that this successful attempt should be ascribed to the sufficient anchoring of the first monolayer of silica particles provided by the contact mechanics adhesion force (polymer layer) and geometrical restriction (physically templated substrates). However, as remarked before, this strategy cannot be employed for polymer particles, as the polymer glue layer is removed at a temperature of 500 °C. On the other hand, García-Santamaría et al.^66^ demonstrated that a pick-and-place approach may be a more promising avenue to develop, albeit extremely slow (≈7 min) for each individual particle in the lattice.

The coassembly process entails using a binary mixture of particles, which is typically prepared in a solution to minimize the impact of surface forces. In dry conditions, however, such mixtures tend to segregate with particle size, type, and density, playing a crucial role in this process.^85^ For instance, when coarse and fine particles are combined in a dry setting, the finer particles often adhere to the surface of larger ones. As such, powder blending under dry conditions is extremely challenging due to cohesive surface forces and triboelectric charging, which would frustrate the coassembly process due to particle clustering.^86^ Most studies in the particle blending field deal with particle diameters of >10 μm (gravity dominated) and processes with a high throughput.^87^ Only a limited number of studies, often in the fields of biology (for instance, particles in inhalers) and alloys (e.g., making superalloys), investigate microparticle mixing. As such, research should focus on blending particle mixtures in order to advance this field. But even if good mixing in dry methods is obtained satisfactorily, it remains to be seen whether ordering is achieved and whether the smaller particles align themselves between the coarser particles.

A way to promote the ordering might be found in two wet assembly techniques that use an external source to initiate binary ordering (Figure 5d,e). In Figure 5d, a 25-layer-thick rock salt crystal is created, consisting of 0.4- and 1.4-μm silica particles using electric field -assisted crystallization.^80^ A high-frequency AC voltage was applied to ensure that the double layer surrounding the particles was not disturbed. In contrast, the amplitude ensured that only the large colloids obtained a significant dipole moment. By manipulating the electric field, different crystals can be grown. Another method is using a combination of magnetic and nonmagnetic particles in solution in conjunction with a structured surface consisting of a nickel grid embedded in PDMS. By placing the surface on a permanent magnet, the otherwise uniform magnetic field is modulated, attracting magnetic particles to the nickel and nonmagnetic particles toward the PDMS (Figure 5e). This method can also discriminate between particles with the same magnetic susceptibility but different sizes because the magnetostatic potential scales with the particle volume. To further demonstrate the accuracy with which the particles can be positioned, a plethora of 3D colloidal molecules is created.

Using external electric or magnetic fields in a template-assisted fashion is an appealing research avenue to pursue in the assembly of binary monolayers without solvents. A significant aspect is that external fields must be strong enough to overcome the existing surface forces among the particles and between the particles and substrates. Regarding the electric field, Jimidar and coworkers have already demonstrated that silica, polystyrene, and PMMA particles can at least be dispersed in air,^20,49^ but this was not sufficient to assemble the beads, as an external vacuum force was needed to assemble a monolayer of particles. It remains to be explored whether the superparamagnetic particles available today can be assembled on a templated surface by using a magnetic field. This will be highly dependent on the magnetic susceptibility of these particles, which heavily depends on the size and concentration of particles.^88^ Thus, it remains to be determined whether the magnetic force is sufficiently strong to promote the ordering of the particles.

A physically templated substrate combined with capillary assembly is also utilized to assemble ternary particle mixtures.^82^ Tuning the depth of the assembly sites (traps) and the surface tension of moving droplets of the suspensions enables the controlled stepwise filling of traps to attain colloidal structures. The structured PDMS template controls the lattice geometry, whereas the filling sequence determines the particle ordering (Figure 5f). Different polystyrene (red, green, blue, and nonfluorescent) combined with plain SiO_2_ particles are assembled with sizes of approximately 1 μm.

To sum up, the formation of binary ordered monolayers has mostly been achieved with wet assembly techniques so far. As demonstrated by Khanh and Yoon^14^ using the solvent-free rubbing method, the layer-by-layer method is promising in attaining ordered binary monolayer structures. However, many of the challenges in tuning the particle–particle interactions discussed in Section 3.1 remain. This may be solved by utilizing a robotic setup to attain binary ordered monolayer structures or even multilayers. A robotic setup can be costly compared to a wet method, but it depends on a trade-off between time efficiency, scale, and quality of the assembled structures needed as well as the cost. On the coassembly side of things, a critical aspect is to achieve a well-dispersed powder mixture, for which the hurdle of optimizing the powder blending process must be solved. As the surface forces are much stronger in dry environments, it is still not guaranteed that ordering will be achieved, as one would need to separate the coarser and finer particles from each other during the assembly process. The use of electric and magnetic fields, as used in wet environments, may be a route to achieving the necessary selectivity.

### 3.3 Shape-Anisotropic Particles

All of the preceding sections have discussed spherical colloidal particles. The unprecedented advancements in polymer and colloidal chemistry to synthesize particles or building blocks with a plethora of different shapes, such as rods,^89^ discs,^90^ tiles,^91^ and dumbbells,^92^ mark the onset of an emerging field of assembling nonspherical, i.e., shape-anisotropic, particles. The assembly of nonspherical particles can be more challenging due to the more complex positional and orientational ordering needed compared to spherical particles. There are different methods to achieve the ordering of nonspherical particles. Similar to the field of binary monolayers (Section 3.2), the assembly of nonspherical particles has mainly been achieved by wet assembly techniques.

Like spherical particles, droplet evaporation in which capillary flow (due to the droplet’s evaporation) drives the assembly process can be exploited to order nonspherical particles. When the particle concentration at the droplet’s rim increases above the crystallization density, it marks the onset of the ordered particle assembly. The contact angle decreases to a critical value due to solvent evaporation, and the contact line recedes, leaving a ring of ordered particles on the surface. An example is shown in Figure 6a,b for nanocubes and nanorods. From these results, it can be inferred that the evaporation droplet technique is highly suitable for assembling small particle dimensions (<300 nm). To stabilize the Au nanoparticles, a molecule was added to the solution, and the ordering is heavily dependent on the concentration of the molecule and the temperature.^89,93^ Particles without the surfactant bilayer tend to aggregate because of the large van der Waals attraction, while a temperature that is too high makes the evaporation process too fast. This balance represents the pitfalls and challenges and may simultaneously be considered the Achilles’ heel of wet assembly.

**Figure 6:**
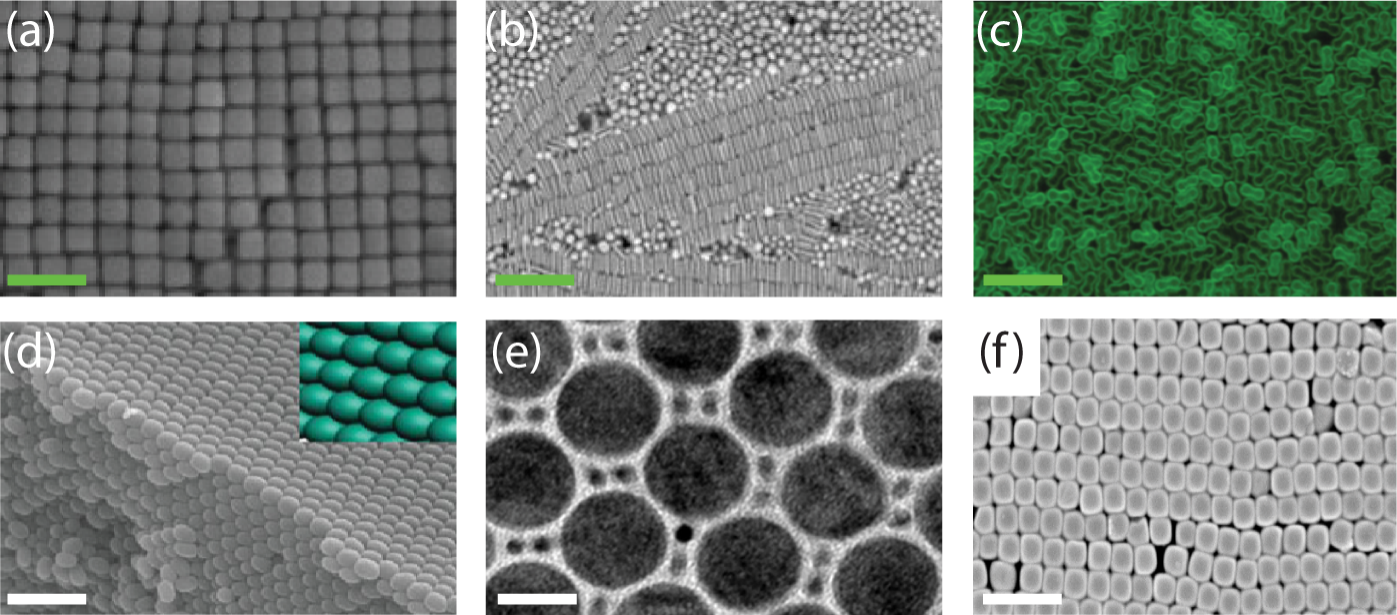
(a) Tetragonally packed Au nanocubes using the droplet evaporation technique, scale bar = 150 nm. Reproduced with permission from ref. 93. Copyright 2008 Wiley. (b) Phase separation between Au nanospheres and nanorods using the droplet evaporation technique, scale bar 200 nm. Reproduced with permission from ref. 89. Copyright 2014 American Chemical Society. (c) Dumbbell-shaped silica particles are assembled using the confined droplet evaporation technique, scale bar = 10 μm. Reproduced with permission from ref. 92. Copyright 2008 Royal Society of Chemistry. (d) 3D assembly of ellipsoidal particles using magnetic vertical deposition technique, scale bar 1.5 μm.^94^ (e) Self-assembled superlattices of nanodisks and nanorods using the liquid–air interfacial assembly, scale bar = 20 nm. Reproduced with permission from ref. 90. Copyright 2015 American Chemical Society. (f) Assembly of 1 μm nanocubes using the unidirectional rubbing method; scale bar 5 μm. Reproduced with permission from ref. 43. Copyright 2021 MDPI.

Adding confinement to the assembly game gives more control over the deposition of the particle using the droplet evaporation technique.^92^ Using a wedge geometry, dense dumbbell particle monolayers are formed due to the alignment of the particles with the walls (Figure 6c). The evaporation-driven self-assembly can be tuned by changing the interaction between the solvent and the substrate or between the particles and the substrates. If the contact angle between the solvent and substrate is higher than the contact angle between the solvent and the particles, the dispersed particles do not form any self-assembled structures at the three-phase contact line.^95^

To further enhance the alignment of particles, external fields can be applied, as shape anisotropy may lead to the preferential orientation of the particle in an external field, which would not be the case for an isotropic, i.e., spherical, particle. Highly ordered particle multilayers of ellipsoidal iron oxide particles are obtained using the vertical deposition technique (as discussed in Section 3.1) in combination with an external magnetic field.^94^ The external magnetic field induced a preferential orientation on the magnetic particles, allowing for precise control of the positional and orientational order of ellipsoidal iron oxide particles in 3D structures (Figure 6d).

Another option for assembling particles is between two liquid interfaces and subsequently allowing them to dry onto a surface (liquid–air interface), which is referred to as emulsion deposition.^91^ First, the crystalline monolayers are formed at the liquid–liquid interface between two immiscible liquids. Second, a third miscible cosolvent is added, which drives the monolayer to the liquid–air interface. As the droplet evaporates, it leaves the ordered monolayer on the surface, enabling the assembly of tiled particles in hexagonal, square, and circular shapes that can be arranged into nicely ordered monolayers.

Using a similar technique, liquid–air interfacial assembly, binary superlattices consisting of nanodisks and nanorods can be made.^90^ In Figure 6e, an example is shown in which the large disks are surrounded by rods (which point out of the image). Different arrangements can be tuned by varying the size ratio between the rods and disks and their concentrations. Using conventional droplet evaporation, phase separation is observed between the differently shaped particles (Figure 6b).^89^ However, this method does not provide the necessary degree of freedom to tweak the process such that ordered monolayers form.

Within the solvent-free assembly realm, only two reports explored the assembly of shape-anisotropic particles, namely zeolite crystal particles on PEI^26^ and silica cubic particles on PDMS,^43^ using the rubbing method (Figure 6f). The latter was performed in an automated fashion,^43^ while the monolayer assembly of the zeolite crystals was achieved through surface functionalization using hydrogen bonding between the bare zeolite crystals and glass substrates through a polymer linker (PEI).^14^ In both cases, the ordering was poor. The poor ordering can be ascribed to the fact that, instead of a rolling motion required for perfect assembly using the rubbing method, the particles slide and behaved unpredictably (similar to a billiard game), disrupting the existing assembly. The drawback of the sliding motion of the silica cubes was corroborated by comparing it to the automated rubbing of spherical silica particles, which resulted in a perfectly ordered monolayer.

Thus, for shape-anisotropic particles such as ellipsoids and dumbbells, a defined rolling axis still allows for controlled monolayer formation, albeit predominantly in one direction. Yet, for particles lacking rotational symmetry, such as squares, pyramids, and disks (although a symmetric axis is present, the tendency to lie parallel to the surface hinders the rotation), rolling is inherently difficult, leading to sliding and disordered arrangements.

The above implies that novel strategies should be developed to overcome the limitations of translating the assembly of shape-anisotropic particles into the dry assembly domain. Introducing structured substrates with localized adhesion control or employing textured stamps may provide a means of influencing particle orientation and promoting order. However, fundamental understanding is lacking regarding whether these nonspherical particles will naturally self-organize due to altered particle–particle and particle–substrate interactions. The latter, in particular, poses a significant challenge, as the larger contact area of nonspherical particles increases adhesion, potentially inhibiting movement and structured assembly.

Furthermore, achieving binary monolayers of nonspherical particles in dry conditions remains an even greater challenge and unexplored territory, with an immense fundamental knowledge gap. Addressing these hurdles will require systematic investigation into adhesion, friction, and geometry interplay in dry assembly processes at the micro- and nanoscale. These limitations elucidate the need for in-depth fundamental research and development of engineering strategies to advance the dry assembly field that can be extended to assembling nonspherical particles, making it a truly versatile method for structuring a broad range of particle shapes and compositions.

## 4 Solvent-Free Assembly Incorporated in Applications

As noted before, the dry rubbing assembly garnered interest over the past decade as a rapid fabrication method for ordered colloidal monolayers. Typically, the dry rubbing assembly can lead to ordered monolayers in <20 s on a 4-in. wafer scale, while wet assembly methods, e.g., Langmuir–Blodgett method, may take a few hours to cover a few centimeters of surface area.^17^ These significant time-scale differences show that solvent-free rubbing assembly is a promising technology for industrial settings. As such, we broadly showcase some applications that have employed the rubbing method to attain ordered structures, providing a perspective to readers that dry-assembled structures can be utilized in a wide breadth of applications. In addition, we shed light on applications that utilized an automated rubbing setup to attain ordered monolayer structures, emphasizing that dry assembly processes may be a cost-effective way for the rapid, mass production of structured or functional surfaces. Moreover, the applications exemplify that dry-assembled monolayers are an inexpensive way for the rapid patterning/structuring of surfaces compared to other fabrication techniques involving expensive lithographic tools, dry etching machines, or complicated chemical procedures.^96^

Superomniphobic polymer films were fabricated by adopting a rubbing process combined with a heating treatment to structure a polymer film on a 4-in. wafer. The dry rubbing assembly of silica microspheres has been shown to be promising compared to other techniques like dip coating or spraying techniques, in attaining polymer films with hexagonally triangular protrusions or hexagonally rectangular micropillars on a large scale,^96^ highlighting the efficacy and efficiency of the solvent-free rubbing method. Similarly, such texturing of polymer films was also explored in the context of ultrasensitive (258.7 kPa^–1^) pressure sensors with an ultralow detection limit (0.68 Pa).^97^ An ordered monolayer comprising 5-μm silica beads was rapidly attained by using the dry rubbing method, serving as a template for structuring polymer films. Pillar-like or needlelike structures were obtained by pouring the desired polymer onto the assembled silica monolayers, which were subsequently treated to obtain the desired structures for the ultrasensitive pressure sensor that demonstrated successful monitoring of large muscle movements and subtle vital signs in humans.^97^

Jimidar et al.^98^ applied the rubbing method to assemble ordered monolayers comprising polymer colloids (500 nm–10 μm) on fluorocarbon-coated Au-covered substrates that were utilized in triboelectric nanogenerators (TENGs) for the first time (Figure 7a). TENGs are small-scale energy-harvesting devices in which ambient waste kinetic energy is converted into electricity via a friction interface. This study established that these monolayers can be rapidly produced (<20 s) on a large scale (25 × 25 mm^2^) using a cost-effective manner to attain microstructured surfaces compared to expensive surface structuring technologies that are commonly exploited to boost the performance of TENG devices. Besides an enhanced output of TENG devices, the authors demonstrated that the performance of these inexpensive granular-interface TENGs was stable over 10,000 cycles, corroborating their robustness. Furthermore, such TENG devices can also be employed to gain insights into the physical phenomena involved in contact electrification or tribocharging of colloidal particles. Due to the possibility of rapid fabrication of the “granular electrodes”, the influence of particle size and composition on the performance could be unraveled, adding to the understanding of the contribution of surface interactions in granular TENGs.

**Figure 7:**
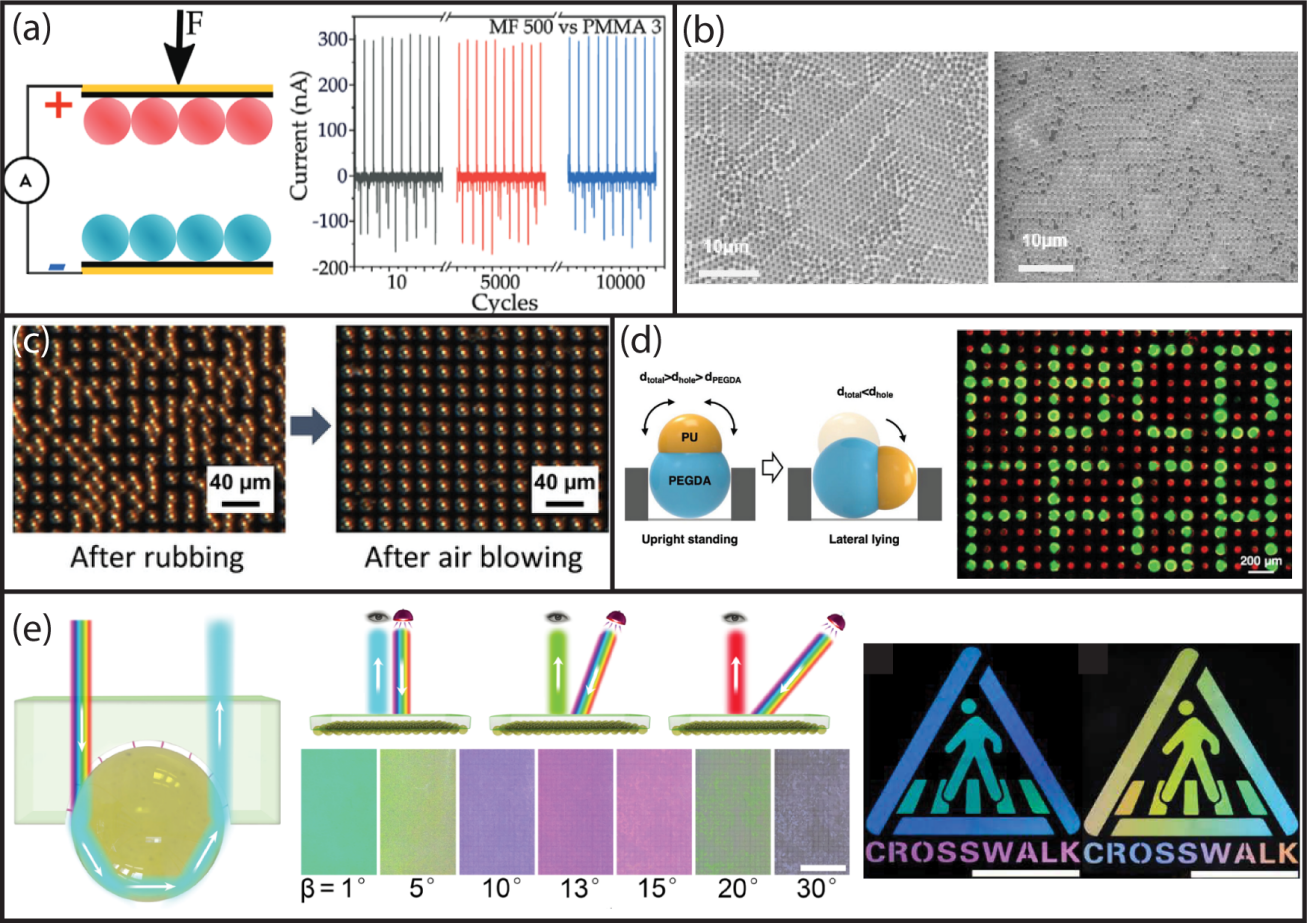
(a) A granular triboelectric nanogenerator (TENG) based on dry assembled particles. The TENG was stable over 10,000 cycles. Reproduced with permission from ref. 98. Copyright 2025 Wiley. (b) Transfer of 1 μm polystyrene particles assembled using the dry rubbing method from an elastomer to a silicon surface, scale bar 10 μm. Reproduced with permission from ref. 17. Copyright 2024 American Chemical Society. (c) A stretchable anisotropic conductive film was created by using the dry rubbing method. Au-coated polystyrene particles are rubbed on a physically templated PDMS sample. The final ordering is achieved by introducing excess pressurized air. Reproduced with permission from ref. 44. Copyright 2024 Wiley. (d) Pixel-based micropatterns based on Janus particles. By using anisotropic particle geometry, different patterns can be assembled. Reproduced with permission from ref. 99. Copyright 2019 Wiley. (e, Left) Schematic illustration of the generation of retroreflective structural color in a single air-cushioned microsphere/polymer bilayer model. (middle) Schematic illustrations (top) and photographs (bottom) of the iridescent colors reflected by an retroreflective structural color film assembled from 15 μm PS microspheres under oblique illumination at an angle β and at a fixed viewing angle of 0°. (Right) Photographs of the traffic sign from 80 and 30 m, demonstrating a smart color-changing visual indication. Reproduced with permission from ref. 100. Copyright 2019 American Association for the Advancement of Science.

As already highlighted, the use of PDMS substrates dominates rubbing assembly research. In terms of colloidal lithography, PDMS substrates are not always suitable.^3,17^ To overcome these limitations, Tzadka et al.^17^ developed an automatic rubbing setup in which an ordered monolayer is assembled between two PDMS sheets and subsequently transferred to a Si or sapphire substrate covered with a PEI layer (Figure 7b). This target PEI layer on the Si or sapphire substrate was used because it electrostatically attracted the colloidal monolayer from the PDMS surface. These monolayers were utilized as a high-throughput nanopatterning technique with periodicity ranging between 200 nm and 2 μm. This bottom-up fabrication approach is used to create nanostructures for antireflective applications and 3D nanostructures for studying the proliferation of T cells, which is potentially significant for cancer immunotherapy. In the latter case, the limitation of expensive and slow e-beam lithography for fast and scalable fabrication of elastomer-based brush arrays to activate T cells is addressed.

To take full advantage of precisely ordered particles, they should remain in position for the intended application in the final product,^101^ even when the geometry of the ordered patterns is arbitrary. This implies that the particles must remain stable without detaching from their position under mechanically strained states, such as bending and stretching, elucidating sufficient adhesion between the particle and substrate. To this end, Park et al.^44^ used physically templated PDMS substrates to precisely position a single Au-coated polystyrene microsphere inside an individual well using an automated rubbing setup. As can be inferred from Figure 7c, excess particles were readily removed by blowing pressurized air, and the adhesion of the particles was promoted by partially cured PEG-DA inside each well of the physically templated PDMS substrate. When a clump of the PEG-DA was missing from the wells, many wells remained empty, underlying that sufficient adhesion is crucial to precisely capture particles, even when using physically templated surfaces. This assembly strategy was developed as a cost-efficient and facile method to produce stretchable anisotropic conductive films that could serve as a soft ionic temperature sensor.^44^

Similar to the assembly of single particles on flexible, physically templated surfaces discussed above, the rubbing method is also explored to assemble programmable positioning of two distinct-sized microparticles.^102^ Given the two particle sizes to be assembled, the larger beads are rubbed first, as they do not fit into the smaller wells, followed by rubbing the smaller particles. This implies that by cleverly engineering the well sizes, different particle sizes can be assembled on the same substrate. This concept was leveraged by the Kim group to assemble Janus particles comprising two lobes of distinct sizes on a templated surface with two different well sizes.^99^ Consequently, the orientation of the Janus particles was affected by the well size during the rubbing assembly, as depicted in Figure 7d. The accurate positioning of the colloids can be noticed from the fluorescence microscopy images displayed in Figure 7d.

Also, in the context of retroreflective structural color films^100,103^ ordered monolayers of polystyrene (PS) microspheres that were attained using the rubbing method have been explored. Cellulose nanocrystals^103^ or the sticky side of transparent tape^100^ were employed to partially embed the particles, i.e., the particles were transferred partially into the polymer film. The ordered PS microspheres act as a retroreflective array, enhancing structural color produced by the polymer film depending on the viewing and illumination angle. Fan et al.^100^ exploited an impressive roll-to-roll process to produce a retroreflective structural color film of 1-m long and 6 cm wide comprising 15 μm PS beads that could be used as a traffic sign.

To sum up, the applications discussed above underscore the broad utility of ordered colloidal monolayers. Notably, solvent-free assembly methods, especially the rubbing technique, are well-suited for automation (though they can be costly if robots are used), enabling precise pixel-based micropatterning. The rubbing method has been demonstrated to achieve order even at a 1-m scale in a roll-to-roll process within a few minutes, whereas such assemblies using wet techniques would take hours. Consequently, dry assembly methods meet the demand for efficient, cost-effective, scalable, and sustainable fabrication processes for mass production.

## 5 Summary and Outlook

As we pursue a more sustainable future, the transition from wet assembly techniques to solvent-free methods offers a promising path forward. Since 2009, the potential of dry assembly has been demonstrated, with an increasing number of studies highlighting its advantages. Over time, a handful of dry assembly techniques have been developed and applied across different fields.

While wet assembly methods still dominate, solvent-free techniques are rapidly gaining traction. For highly ordered and confined particle monolayers, dry methods can achieve precision and quality comparable to, if not better than, their wet counterparts; yet they do so with greater simplicity, speed, scalability, and environmental benefits by eliminating solvents (cf. Table 1). Moreover, dry rubbing offers a distinct advantage when using well-like structures as physical templates: it accommodates size and shape variations more effectively, leading to higher packing densities. However, the use of external stimuli such as electric and magnetic fields remains limited in dry environments as they should induce strong forces to surpass the significant surface interaction forces at small particle sizes (<10 μm). As such, a profound fundamental understanding and better control of these forces are essential to unlock the full potential of externally guided dry assembly.

**Table 1:** Overview of a Comparison between Dry and Wet Assembly Methods in Terms of Various Indicators, Highlighting Challenges and Opportunities

	Dry Assembly	Wet Assembly
Interactions	Capillary, van der Waals, contact mechanics, electrostatics (tribocharging)	van der Waals, electrostatics
Structures	2D; 3D (scarce)	2D; 3D
Conditions	Humidity, (tribo-)charging, elasticity of particles and substrates	Particle properties, pH temperature, surface tension
Time	Rapid (<20 s)	Min—h (length scale dependent)
Versatility	Broad spectrum of particle sizes and types	Adapt based on particle size and type
Scalability	4-in. wafer/1-m long	4-in. wafer
Size	Rubbing (≈20 nm—tens of μm), electric field (3 μm), acoustic field (40 μm)	A few tens of nanometers—μm
Shapes	Spherical (mostly)	Spherical and shape-anisotropic

The challenges become more pronounced when dealing with ordered multilayers, binary layers, and nonspherical particles. Here, wet techniques remain dominant due to the absence of viable dry alternatives. Strong surface interactions and a lack of control over these forces hinder progress in dry assembly. In multilayer and binary layer formation, the reduced particle–substrate interaction in the second layer and deviations from a flat surface compromise stability, disrupting the assembly process. However, advancements made in wet assembly can guide new strategies, such as enhancing particle–substrate adhesion, to enable solvent-free methods for three-dimensional crystals.

The assembly of nonspherical particles poses an even greater challenge due to their movement and arrangement complexity. Unlike spherical particles, which can roll predictably, nonspherical particles (e.g., ellipsoids, pyramids, and disks) exhibit asymmetric rolling behavior, or no rolling capability at all, making dry assembly unpredictable. This fundamental limitation could be addressed through alternative approaches, such as robotic pick-and-place techniques,^72^ which bypass the need for rolling altogether.

Despite being a relatively young field, dry assembly techniques, particularly the rubbing method, have already found their way into applications such as triboelectric nanogenerators, single-pixel flexible displays, and antireflective coatings. Their efficiency, low cost, and scalability (4-in. wafer and even on a 1-m-long plate) make them an attractive alternative to traditional wet assembly. In terms of monolayer assembly, the dry methods seem to outperform the wet assembly methods when it comes to the time and length scale (wafer scale or more) of the assembled monolayers. As such, this technology can be translated to a roll-to-roll process in industry, particularly as automated rubbing has been recently reported by different groups.^17,43,44^

With this perspective, we urge further research to bridge the fundamental knowledge gap in dry assembly, particularly in understanding surface interactions at the micro- and nanoscale. Expanding this field will not only enhance the ability to manipulate particles with greater precision but also establish solvent-free assembly as a viable, scalable, and sustainable alternative for a wide range of applications. Additionally, developing novel methods is crucial for advancing the field, enabling innovative approaches that overcome current limitations and unlock new possibilities in dry particle assembly. By deepening our understanding and expanding the toolkit of available techniques, we can propel solvent-free assembly from a promising alternative to a fully developed and widely adopted approach, paving the way for a more sustainable future. Besides the sustainability aspects, we envision that the robotic automation potential of solvent-free assembly methods is expected to create a surge in integrating such methods to gain time and cost efficiency in fabricating ordered micro- and nanostructures. While an automated robotic system may seem to be an expensive avenue, it will come down to a trade-off between costs, time efficiency, precision, and scale needed for the assembly process.
